# The equilibrium of tumor suppression: DUBs as active regulators of PTEN

**DOI:** 10.1038/s12276-022-00887-w

**Published:** 2022-11-16

**Authors:** Audrey Christine, Mi Kyung Park, Su Jung Song, Min Sup Song

**Affiliations:** 1 Soonchunhyang Institute of Medibio Science, Soonchunhyang University, Cheonan-si, Chungcheongnam-do 31151 Republic of Korea; 2 Department of Integrated Biomedical Science, Soonchunhyang University, Cheonan-si, Chungcheongnam-do 31151 Republic of Korea; 3 Department of Molecular and Cellular Oncology, The University of Texas MD Anderson Cancer Center, Houston, TX 77030 USA

**Keywords:** Cancer, Ubiquitylation

## Abstract

*PTEN* is among the most commonly lost or mutated tumor suppressor genes in human cancer. PTEN, a bona fide lipid phosphatase that antagonizes the highly oncogenic PI3K-AKT-mTOR pathway, is considered a major dose-dependent tumor suppressor. Although PTEN function can be compromised by genetic mutations in inherited syndromes and cancers, posttranslational modifications of PTEN may also play key roles in the dynamic regulation of its function. Notably, deregulated ubiquitination and deubiquitination lead to detrimental impacts on PTEN levels and subcellular partitioning, promoting tumorigenesis. While PTEN can be targeted by HECT-type E3 ubiquitin ligases for nuclear import and proteasomal degradation, studies have shown that several deubiquitinating enzymes, including HAUSP/USP7, USP10, USP11, USP13, OTUD3 and Ataxin-3, can remove ubiquitin from ubiquitinated PTEN in cancer-specific contexts and thus reverse ubiquitination-mediated PTEN regulation. Researchers continue to reveal the precise molecular mechanisms by which cancer-specific deubiquitinases of PTEN regulate its roles in the pathobiology of cancer, and new methods of pharmacologically for modulating PTEN deubiquitinases are critical areas of investigation for cancer treatment and prevention. Here, we assess the mechanisms and functions of deubiquitination as a recently appreciated mode of PTEN regulation and review the link between deubiquitinases and PTEN reactivation and its implications for therapeutic strategies.

## Cancer: Reactivating a tumor suppressor

Studying ways to reactivate a tumor-suppressing protein called PTEN may help in finding new cancer therapies. PTEN represses a signaling pathway that, when over-activated, strongly influences cells to become cancerous. Although some cancers are caused by mutations of PTEN itself, new research shows that proteins called deubiquitinases (DUBs), which regulate stability and activity of target proteins, can turn on and off PTEN activity and cancer. Su Jung Song at Soonchunhyang University, Cheonan-si, South Korea, and Min Sup Song at The University of Texas, Houston, USA, and coworkers have reviewed how DUBs can affect PTEN, reporting on the molecular mechanisms of cancer-specific DUBs, and highlighting the potential to pharmacologically target DUBs in order to boost PTEN activity and suppress cancer development. This review illuminates a promising new approach to developing therapies for several types of cancer.

## Introduction

**Fig. 1:**
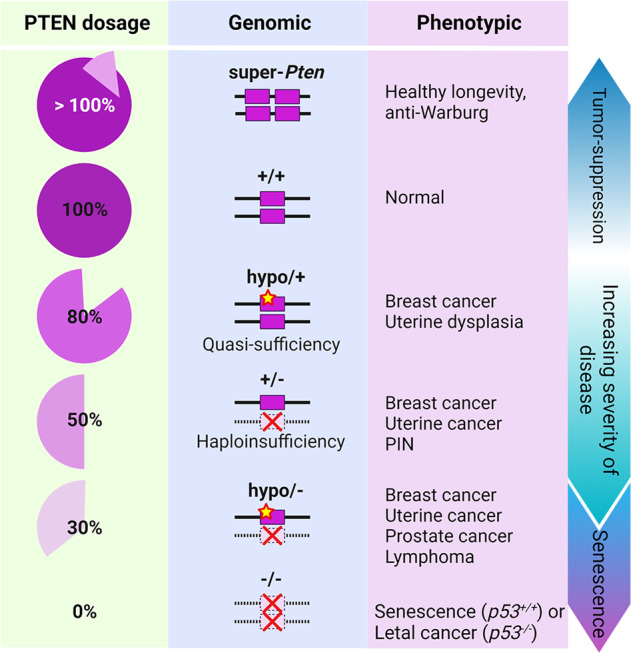
The PTEN continuum in tumor suppression. PTEN function can be compromised via genetic disruption, which results in a stepwise loss of PTEN (50% or 100%). Posttranslational modifications, including ubiquitination and deubiquitination, of PTEN can fine-tune PTEN functionality via a continuum of tumor suppression. Notably the phenotypes acquired throughout the continuum of functional PTEN loss are differentially manifested depending on tissue type.

*PTEN* (*p*hosphatase and *ten*sin homolog deleted on chromosome 10) is one of the most frequently lost or mutated tumor suppressor genes in human cancer^1,2^. Its encoded protein, PTEN, negatively regulates the phosphoinositide 3-kinase (PI3K)−AKT−mammalian target of rapamycin (mTOR) signaling pathway through dephosphorylation of the plasma membrane lipid phosphoinositide-3,4,5-triphosphate. As a consequence, loss of PTEN function leads to potent derepression of the PI3K-AKT pathway, which stimulates cell survival, proliferation, energy metabolism, and architecture^3,4^. PTEN also shows protein phosphatase, specifically dephosphorylating tyrosine-, serine- and threonine-phosphorylated polypeptides in vitro^5^ and several different cellular substrates, including focal adhesion kinase (FAK), cAMP-responsive element-binding protein (CREB), tyrosine kinases SRC and PTK6, and insulin receptor substrate 1 (IRS1)^6–10^. Furthermore, phosphatase-independent activities (mostly scaffolding) of PTEN regulate many processes, such as DNA replication, DNA repair, genomic stabilizing events, and cell cycle progression, have also been identified^11–14^, implicating the noncanonical roles of PTEN in tumorigenesis. Germline heterozygous pathogenic mutations in *PTEN* have been described in a variety of rare syndromes with different clinical presentations that are collectively known as PTEN hamartoma tumor syndromes (PHTSs), which exhibit features of both benign and malignant tumors^15^. Many modeling efforts with *Pten*-knockout mice have demonstrated that PTEN functions in a haplo-insufficient manner^16–18^; paradoxically, when PTEN levels are nearly completely loss, a strong cellular senescence program is triggered^19,20^, which is a ‘fail-safe’ brake on tumor progression^21^. Notably, an analysis of a series of mouse models of hypomorphic *Pten* has revealed the tremendous functional consequences of a subtle reduction in PTEN protein levels^22,23^, which can promote cancer susceptibility and favor tumor progression. Additionally, increased PTEN levels in transgenic models result in viable mice displaying a tumor-resistant, anti-Warburg metabolic state^24,25^. Thus, PTEN plays a critical dose-dependent role in tumor suppression, and therefore, understanding the regulatory mechanisms that fine-tune PTEN activity has become a paramount therapeutic goal (Fig. 1).

Although PTEN function can be compromised by genetic mutations in inherited syndromes and sporadic cancers, posttranslational modifications (PTMs) of PTEN can play key roles in the dynamic regulation of its activity and function. For example, phosphorylation of PTEN affects protein stability and activity^26,27^. PTEN can also be SUMOylated, which increases its nuclear retention, thereby supporting its nuclear function in DNA repair mechanisms^11,28^. Ubiquitination requires the concerted action of activating (E1)-conjugating (E2)-ligating (E3) enzymes^29^. HECT-type E3 ubiquitin ligases, including neuronal precursor cell-expressed developmentally downregulated 4-1 (NEDD4-1) and WW domain-containing E3 ubiquitin protein ligase 1 (WWP1) and 2 (WWP2), have been shown to converge at ubiquitination-mediated PTEN regulation^30^; specifically, PTEN monoubiquination leads to either PTEN translocation to the nucleus or exosomal transport (by NEDD4-1)^31,32^, while PTEN polyubiquitination suppresses its stabilization (NEDD4-1 and WWP2)^33,34^ or dimerization and subsequent membrane recruitment (WWP1)^25,35^.

**Fig. 2:**
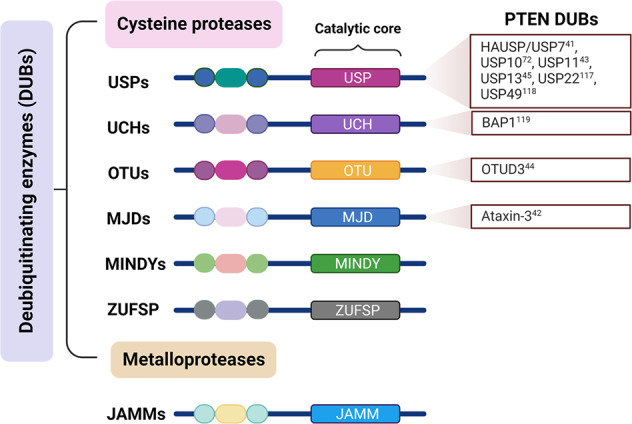
Schematic diagram of the domain architecture of DUBs. Two classes of proteases (cysteine proteases and metalloproteases) are DUBs, with most DUBs cysteine proteases. Cysteine protease DUBs can be classified into six subfamilies based on their DUB domains: USP, UCH, OTU, MJD, MINDY, and ZUFSP. Metalloprotease DUBs include a JAMM DUB domain. USP, ubiquitin-specific protease; UCH, ubiquitin C-terminal hydrolase; OTU, ovarian tumor protease; MJD, Machado–Joseph disease protease; MINDY, motif interacting with Ub-containing novel DUB family; ZUFSP, zinc finger with UFM1-specific peptidase domain; JAMM, JAB1/MPN/Mov34 metalloenzyme.

Increasing evidence has shown that deregulated deubiquitination leads to detrimental effects on PTEN levels and subcellular partitioning to promote tumorigenesis. Deubiquitinating enzymes (DUBs) are proteases that deconjugate ubiquitin from ubiquitinated substrates and thereby remodel polyubiquitin chains on target proteins to counteract the protein ubiquitination mediated by E3 ubiquitin ligases^36^. DUBs are categorized into two major classes, cysteine proteases and metalloproteases. The former class includes six main superfamilies^36^: ubiquitin-specific protease (USP), ubiquitin C-terminal hydrolase (UCH), ovarian tumor protease (OTU), Machado–Josephin domain (MJD) protease, and the recently discovered MINDY^37^ and ZUFSP^38,39^ families (Fig. 2). The JAB1/MPN/MOV34 (JAMM)-motif proteases bind to zinc and therefore are classified as metalloprotease-type DUBs^40^. Indeed, HAUSP/USP7, Ataxin-3, USP10, USP11, USP13, and OTUD3 have all been identified as PTEN DUBs; HAUSP specifically removes monoubiquitin from PTEN to promote its nuclear export^41^; Ataxin-3 restricts PTEN transcription^42^; and USP10, USP11, USP13 and OTUD3 increase PTEN stability in different cancer-specific contexts^43–45^. As ongoing research reveals the precise molecular mechanisms by which cancer-specific deubiquitination of PTEN regulates its roles in the pathobiology of cancer, the ability to pharmacologically modulate or otherwise counteract specific DUBs of PTEN, both selectively and in combination, is becoming a critical area of investigation for cancer prevention and treatment. In this review, we summarize the pathological and functional mechanisms of PTEN DUBs and describe how their functions dictate cancer cell biology and physiology while highlighting opportunities for therapeutic intervention.

## Herpesvirus-associated ubiquitin-specific protease (HAUSP)/Ubiquitin-specific protease 7 (USP7)

HAUSP (also known as USP7) was first identified as a protein that binds herpes simplex virus E3, ubiquitin ligase ICP0, and Epstein‒Barr virus nuclear antigen 1 (EBNA1)^46^, indicating its relevance in key cellular processes important in viral infection. All USPs share a conserved catalytic core, while their unique substrate specificity is determined by various accessory substrate-binding domains tethered to a catalytic domain^36^ (Fig. 2). HAUSP contains an NH2-terminal tumor necrosis factor receptor-associated factor (TRAF)-like domain, a central catalytic core, and five ubiquitin-like (UBL) domains in the COOH terminus^47^. The TRAF-like domain in HAUSP can recognize the P/AxxS motifs shared by all TRAF-like domain-binding substrates, including EBNA1, the tumor suppressor p53, the ubiquitin E3 ligase MDM2 (mouse double minute 2), and the MDM2 homolog MDM4^48^. In overexpression experiments, HAUSP has been shown to bind to, deubiquitinate, and stabilize p53^49^, whereas disruption of *HAUSP* expression in human cells and transgenic mice resulted in acquisition the opposite phenotypes, leading to stabilization and functional activation of p53 due to the destruction of MDM2^50,51^, which suggests a dynamic role for HAUSP in the p53–MDM2 pathway. The C-terminal UBL domains can regulate the activation and specificity of HAUSP^52^ and function as additional platforms for substrate binding to highly basic motifs (R/KxKxxxK) within its substrates, including ICP0, UHRF1, DNMT1 and RNF169^53–55^.

**Fig. 3:**
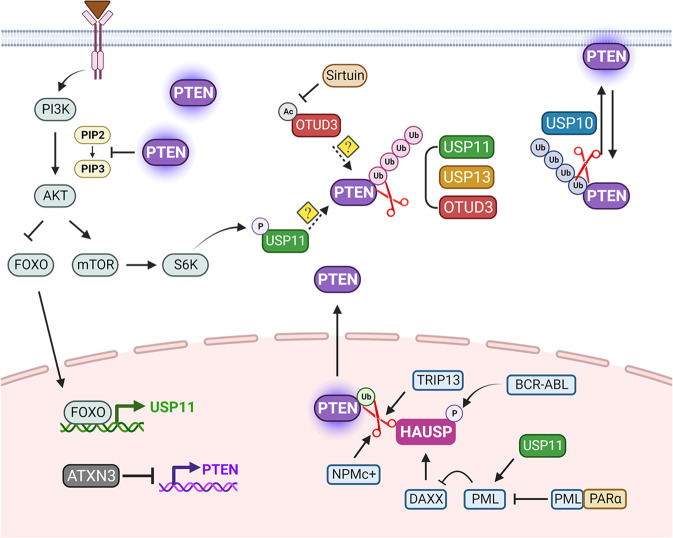
Proposed model showing the mechanisms of DUB action for PTEN. While HAUSP/USP7 induces deubiquitination and subsequent nuclear exclusion of monoubiquitinated PTEN in the nucleus, where it can control the cell cycle and genomic stability, PML-RARα, NPMc+, and BCR-ABL promote HAUSP-mediated PTEN deubiquitination in blood-borne cancers. USP11 plays a role in the maintenance of the effective levels of both nuclear and cytosolic PTEN for tumor suppression, and interestingly, its expression and activity are regulated by the PTEN/PI3K pathway. Furthermore, in the cytoplasm, USP11, USP13, and (acetylated) OTUD3 catalyze the removal of the K48-linked polyubiquitin chain on PTEN to enhance protein stability, whereas USP10 recognizes and removes the K63-linked polyubiquitin chain from PTEN, leading to PTEN recruitment to the plasma membrane. Ataxin-3 represses PTEN by inhibiting its transcription. PTEN phosphatase and tensin homolog deleted on chromosome 10, HAUSP herpesvirus-associated ubiquitin-specific protease, USP10 ubiquitin-specific protease 10, USP11 ubiquitin-specific protease 11, USP13 ubiquitin-specific protease 13, OTUD3 OTU deubiquitinase 3, PI3K phosphoinositide 3-kinase, PIP2 phosphoinositide-4,5-biphosphate, PIP3 phosphoinositide-3,4,5-triphosphate, mTOR mammalian target of rapamycin, PML promyelocytic leukemia, NPMc+ cytoplasmic nucleophosmin, TRIP13 thyroid hormone receptor-interacting protein 13.

HAUSP was identified as the first bona-fide PTEN deubiquitinase^41^ (Fig. 3) and can interact with PTEN both in vitro and in vivo. The domains of HAUSP critical for binding PTEN have not been determined, but the PTEN protein contains four P/AxxS motifs and an R/KxKxxxK motif; therefore, it will be interesting to identify the domain(s) of HAUSP that bind PTEN, the true PTEN sequence recognized by HAUSP, and the nature of their interactions. Although the E3 ligase NEDD4-1 (and/or additional E3s) monoubiquitinates PTEN at lysine residues 13 and 289 for its nuclear import^32,56,57^, HAUSP overexpression can lead to the deubiquitination of the monoubiquitinated PTEN protein and to subsequent nuclear exclusion of PTEN^41^. Notably, this phenotype is associated with more aggressive cancers, implying that when aberrantly expressed, HAUSP is an oncogene, functioning through its ability to disrupt PTEN function. Indeed, HAUSP is overexpressed and associated with unfavorable prognosis in many different types of human cancers, including brain, breast, cervical, lung, prostate, skin, stomach, and hemopoietic cancers^41,54,58–63^, and high HAUSP expression and PTEN nuclear exclusion are strongly and positively correlated in human cancers^41,58,64,65^. Intriguingly, various regulatory mechanisms in cancer influence the propensity of HAUSP to mediate PTEN deubiquitination. For example, in leukemias and prostate cancer, promyelocytic leukemia (PML) plays a critical regulatory role by inhibiting HAUSP activity through death domain-associated protein (DAXX), which in turn favors PTEN nuclear localization^41^. Similarly, nucleophosmin/B26 counteracts HAUSP-mediated deubiquitination and subsequent shuttling of PTEN to the cytoplasm^65^, supporting the notion that PTEN is delocalized in acute myeloid leukemia with mutated nucleophosmin (e.g., NPMc+). In contrast, BCR-ABL and thyroid hormone receptor-interacting protein 13 (TRIP13) enhance deubiquitination and nuclear exclusion of PTEN through activation of HAUSP in chronic myeloid leukemia and multiple myeloma, respectively^66,67^. These clinical and functional studies suggest that aberrant activation or overexpression of HAUSP may promote tumorigenesis, making HAUSP a target for therapeutic intervention in strategies to restore normal PTEN localization and tumor-suppressive function, as we discuss further below.

## Ubiquitin-specific protease 10 (USP10)

USP10 is a deubiquitinase involved in diverse cellular processes, including the DNA damage response, metabolic homeostasis, and ribosome recycling. Upon DNA damage, USP10 accumulates in the nucleus, where it is phosphorylated by ATM kinase, and subsequently deubiquitinates p53^68^. USP10 also interacts with deubiquitinates and enhances the activity of the master energy-sensor AMP-activated protein kinase-α (AMPKα)^69^. Furthermore, USP10 can deubiquitinase Beclin1, a key promoter of autophagy, and protect it from degradation, thus promoting autophagy^70^. Interestingly, Beclin1 also controls the protein stability of USP10 by regulating its deubiquitinating activity, forming a feedback loop. Similarly, USP10 prevents lysosomal degradation of 40 S subunits of ribosomes and ensures ribosome recycling associated with autophagy^71^. Given the importance of the energy balance and autophagy in metabolic disease, USP10 may represent a potential drug target for metabolic syndrome. Furthermore, the role of USP10 in cancer has recently been expanded to include deubiquitinase activity for PTEN. Indeed, USP10 restores the membrane localization and phosphatase activity of PTEN by reversing the tripartite motif-containing 25 (TRIM25)−mediated K63-linked polyubiquitination found in lung cancer^72,73^. Since USP10 is frequently downregulated in human cancers, including lung, gastric, colorectal, and small intestinal carcinomas^74,75^, restoration of USP10 function may represent a new therapeutic strategy for cancer prevention and treatment through PTEN reactivation.

## Ubiquitin-specific protease 11 (USP11)

*USP11* was originally identified as one of inherited X-linked retinal disorder genes at Xp11.23^76^, although a common deletion within the *USP11* interval had been also found in ovarian cancer^77^. X-linked tumor suppressor genes are potentially significant to tumorigenesis because they can be functionally silenced by loss of heterozygosity or mutation of a single allele^78^, and skewed X inactivation may lead toward or against disease^79^. Interestingly, a recent and extensive review described USP11 as a predictive and prognostic factor in human cancers of various histologies^80^; indeed, USP11 is often repressed in brain, breast, skin, and prostate cancers but upregulated in colorectal and hepatocellular carcinomas^80^. As a deubiquitinase, USP11 interacts with multiple substrate proteins linked to cancer-related pathways. For example, USP11 recruits BRCA1 to chromatin by deubiquitinating PALB2 (partner and localizer of BRCA2) in a cell cycle-dependent manner or stabilizing MYCN in neuroblastoma^81^. Notably, USP11 has been found to be rapidly lost after DNA damage in a manner dependent on ATM/ATR induction. In brain tumors, USP11 deubiquitinates and stabilizes PML, and its transcription is inhibited by the Notch effector Hey1^82^. USP11 can also regulate immune cell differentiation by deubiquitinating and stabilizing the NF-κB inhibitor IκB^83^. However, there was insufficient direct genetic evidence to determine the precise role of USP11 in tumorigenesis.

More recently, we found that while mice lacking *Usp11* displayed increased susceptibility to PTEN-dependent tumor initiation, growth and metastasis, USP11 antagonized PI3K/AKT activity by reversing polyubiquitination and subsequently upregulating PTEN expression both in vitro and in vivo^43^, revealing it to be both an X-linked tumor suppressor and an important physiological PTEN deubiquitinase (Fig. 3). The downregulation of USP11 in breast, kidney, thyroid and prostate cancers is closely related to PTEN protein instability, regulating the occurrence and progression of these cancers, and is correlated with worsened prognosis^43,84^. USP11 also acts as an indicator of cell density, thereby controlling the physiological dose of PTEN. Furthermore, we discovered that PTEN-induced FOXO activation promotes *USP11* transcription, which in turn stabilizes PTEN, suggesting that PTEN autoregulates itself through a PI3K-FOXO-USP11 feedforward loop to create a PTEN ‘integrated circuit’ that induces tumor suppression. Similarly, the activity of USP11 is regulated by phosphorylation mediated by PI3K-AKT-mTOR-S6 kinase signaling in diffuse large B-cell lymphoma^85^. Determining whether S6K-phosphorylated USP11 modulates PTEN levels will, however, require further investigation. Interestingly, both PTEN and USP11 participate in the homologous recombination (HR) DNA repair pathway^12,86^, and their deficiency results in hypersensitivity to PARP inhibition^86,87^. Further work is needed to precisely characterize the function/activity of the USP11-PTEN axis in DNA repair by the HR pathway to maintain genomic stability and suppress tumorigenesis.

## Ubiquitin-specific protease 13 (USP13)

*USP13* is localized adjacent to *PIK3CA* in the 3q26.3 locus, which is frequently amplified in human cancers such as brain, lung, ovarian, esophageal and cervical cancers, and high USP13 expression is correlated with poor survival outcomes^88–91^. In ovarian tumors, upregulation of USP13 enhances deubiquitination and stabilization of ACLY (ATP citrate lyase) and OGDH (oxoglutarate dehydrogenase), two key enzymes that drive glutaminolysis, ATP generation, and lipid synthesis in cancer metabolism^89^, and MCL1, a pivotal member of the antiapoptotic BCL-2 family of proteins^91,92^. As an oncogene, USP13 stabilizes c-Myc by antagonizing FBXL14-mediated ubiquitination to maintain glioma stem cell stemness, establish tumorigenic potential and promote cholangiocarcinoma nucleotide metabolism^88,93^. As previously mentioned, USP10 mediates the deubiquitination of Beclin1, and USP13 can directly regulate the deubiquitination of USP10 to promote the formation of autophagosomes^70^. USP13 can deubiquitinate RAP80 (receptor-associated protein 80) and promote the recruitment of the RAP80-BRCA1 complex to damage sites, fine-tuning the DNA repair system^94^. USP13 has also been identified as ERAD E3 ligase gp78-associated deubiquitinase for Ubl4A, a component of the ERAD chaperone complex, and thus promotes ER quality control^95^. Importantly, overexpression of *Usp13* accelerates tumorigenesis, enhances tumor metastasis, and causes poor outcomes in transgenic mouse models of ovarian cancer^96^, underscoring its importance in promoting tumorigenesis in vivo. In addition to its oncogenic roles, USP13 exerts a tumor-suppressive role by deubiquitinating PTEN in different types of cancers. For example, overexpression of USP13 blocks the AKT signaling pathway and suppresses tumor cell proliferation, invasion, and glycolysis by upregulating PTEN, while USP13 levels are downregulated in breast, bladder, and oral squamous tumors, in correlation with PTEN levels^45,97,98^ (Fig. 3). These studies suggest that USP13 plays context-dependent oncogenic and tumor-suppressive roles and that up- or downregulation of USP13 and its target substrates/pathways can contribute to tumorigenesis.

## OTU domain-containing protein 3 (OTUD3)

By regulating the deubiquitination of diverse key substrate proteins, the OTU (ovarian tumor protease) family member OTUD3 plays an important role in the processes of innate antiviral immunity, metabolism homeostasis, and tumorigenesis. OTUD3 has been identified as an acetylation-dependent deubiquitinase that restricts innate antiviral immune signaling^99^. Mechanistically, acetylation of the core OTU domain in OTUD3 markedly enhances deubiquitinase activity on MAVS (mitochondrial antiviral-signaling protein), thereby inhibiting the innate antiviral immune response. Upon viral infection, sirtuin 1 (SIRT1) is recruited to deacetylate OTUD3, leading to the inactivation of OTUD3, which relieves MAVS suppression. OTUD3 has also been recently implicated in the control of energy metabolism^100^. While OTUD3 regulates various genes involved in glucose and lipid metabolism by deubiquitinating and stabilizing peroxisome proliferator-activated receptor-delta (PPARδ), *Otud3*-deficient mice fed a high-fat diet developed greater obesity, dyslipidemia, and insulin resistance, suggesting that aberrant OTUD3 expression may be associated with obesity and a high risk of diabetes.

Emerging evidence has suggested cancer-associated functions of OTUD3 in multiple types of human cancer. For example, OTUD3 interacts with the ZFP36 ring finger protein through its OTU region and stabilizes it by inhibiting FBXW7-mediated ubiquitination, which in turn induces *VEGF-C* mRNA decay to prevent lymphatic metastasis of human esophageal cancer^101^. Furthermore, OTUD3 has been identified as a potent deubiquitinase for PTEN and thus a tumor suppressor in breast cancer (Fig. 3). OTUD3 (OTU region) directly interacts with PTEN (C2 domain), deubiquitinating and stabilizing the PTEN protein to suppress PI3K-AKT signaling^44^. OTUD3 transgenic mice exhibit higher PTEN expression and show a reduced tendency for breast cancer tumorigenesis. The reduction in OTUD3 expression, concomitant with decreased PTEN protein levels, correlates with breast cancer aggressiveness and poor prognosis. As the full activation of OTUD3 may require its acetylation^99,100^, it will be interesting to determine whether OTUD3 acetylation is also involved in PTEN regulation. Nevertheless, an intriguing puzzle has been suggested following a recent study of the accelerated development of lung carcinomas after deletion of *Otud3* in mice^102^. In contrast to its level in breast cancer, *OTUD3* is highly expressed in human lung cancer, and its upregulation is associated with unfavorable prognoses. Furthermore, in lung cancer, OTUD3 fails to regulate PTEN and, in contrast, maintains the stability of the oncoprotein GRP78 (glucose-regulated protein 78-kDa), showing the tumor tissue complexity of the functional role of played by a given deubiquitinase. These findings suggest that future studies should optimize the accurate stratification of deubiquitinase-targeted therapies for specific organs or tissues.

## Ataxin-3 (ATXN3)

Machado–Joseph disease (MJD, also known as spinocerebellar ataxia type 3 or SCA3) is the most common dominant ataxia in the world and is caused by abnormal expansion of CAG repeats in a coding region of *ATXN3*, which produces an elongated polyglutamine (polyQ) tract in the Ataxin-3 protein^103^. Ataxin-3 contains an NH2-terminal ubiquitin-protease (Josephin) domain and COOH-terminal polyQ stretch and ubiquitin-interacting motifs^103^. As a deubiquitinase, Ataxin-3 plays a role in protein quality control and DNA repair by deubiquitinating several essential substrates, including the neuroprotective E3 ligases Parkin and CHIP^104,105^ and the DNA damage response and repair mediators p53, MDC1, RNA polymerase II, and CHK1^106^. A small interfering RNA (siRNA) screen for deubiquitinases revealed that three MJD subfamily members, including Ataxin-3, can inhibit PTEN expression^42,107^. However, Ataxin-3 regulates PTEN transcript abundance but not protein stability, suggesting that its role is independent of direct PTEN deubiquitination.

## Targeting PTEN DUBs for cancer therapy

PTEN is a bona fide lipid phosphatase that opposes the activation of the highly oncogenic PI3K-AKT-mTOR pathway and is considered a major dose-dependent tumor suppressor. While PTEN itself is not considered a ‘druggable’ target, the pathological mechanisms that modulate PTEN protein levels and activity offer possible routes for cancer therapy. Furthermore, the predominant genetic change associated with loss of function is deletion of only a single gene copy of *PTEN*, underscoring the importance of targeting the nongenomic mechanisms of PTEN loss of function for the prevention and treatment of cancer. Along with the previously mentioned biological and clinical relevance of PTEN DUBs in tumorigenesis, PTEN DUBs may represent promising targets for therapeutic PTEN reactivation regimens in many types of cancer. Therefore, the activity of PTEN DUBs can likely be pharmacologically manipulated to fully reactivate PTEN, resulting in new and innovative approaches to the prevention and treatment of cancer (Table 1). Indeed, a small-molecule inhibitor of HAUSP/USP7, P5091, has been shown to restore the monoubiquitination and nuclear localization of endogenous PTEN and to induce cell growth arrest and apoptosis in blood-born cancers^58,66^. In addition to PTEN, p53 is upregulated by P5091, but its cytotoxic activity is not dependent on p53^108^. Other recently developed (pre)clinical HAUSP inhibitors (e.g., FT671, XL188, and GNE6640)^109–111^ will need to be used to establish a portfolio of HAUSP-PTEN axis-targeting drugs for use in future cancer therapies. Additionally, successful PTEN reactivation through disruption of the PML-DAXX-HAUSP complex by Trisenox (arsenic trioxide), which is currently used to treat patients with acute promyelocytic leukemia^41^, may pave the way to clinical trials for prevention and therapy for solid tumors at large.

**Table 1:** Therapeutic potential of targeting PTEN DUBs.

DUBs	Compounds	Effects on DUBs	Effects on PTEN	References
USP7	P5091	Inhibition	Nuclear localization	^58,66^
	FT671, FT827	Inhibition	ND	^109^
	XL188	Inhibition	ND	^110^
	GNE6640, GNE6776	Inhibition	ND	^111^
	Compound 2, 4, 5	Inhibition	ND	^120^
	HBX-19818, HBX-28258	Inhibition	ND	^121^
USP10	Spautin-1	Inhibition	ND	^70^
	Metformin	Activation	ND	^112^
USP11	Mitoxantrone	Inhibition	ND	^122^
	Resveratrol	Activation	Stability	^43^
	Psammaplysene A	Activation	Stability	^43^
USP13	Spautin-1	Inhibition	ND	^70^
OTUD3	Rolapitant	Inhibition	ND	^123^
	Ex-527	Activation	ND	^124^
Ataxin-3	Eeyarestatin-1	Inhibition	ND	^125^

Given the significance of USP10, USP11, USP13 and OTUD3 in PTEN stability, the development of a potent PTEN activation approach through manipulation of these DUBs may represent an attractive strategy for cancer prevention and treatment. For example, as AMPK-mediated phosphorylation of USP10 enhances USP10 activity^69^, treatment with metformin, which is used clinically to activate AMPK^112^, can lead to the upregulation of USP10 and, thus, PTEN-induced tumor suppression. In addition, resveratrol and psammaplysene A have been found to induce *USP11* transcription mediated through FOXO and, in turn, appreciably elevate PTEN protein levels by increasing PTEN deubiquitination and hence PTEN stability^43^. Thus, new stratifications based on up- and downregulation of PTEN DUBs can ensure PTEN protein localization and activity and optimize PTEN DUB-targeted therapies, which may be specifically tailored to human cancers that do not exhibit homozygous (biallelic) loss of *PTEN*.

## Concluding remarks

PTEN antagonizes the oncogenic PI3K-AKT-mTOR signaling pathway, which is frequently activated in cancers. *PTEN* deletions are often found in more aggressive tumors and are associated with worsened prognosis, increased tumor metastases, and a greater chance of recurrence after treatment. Emerging evidence has also shown that, similar to the genomic disruptions that inactivate a given *PTEN* allele, ‘nongenomic’ pathological mechanisms that reduce PTEN protein levels and activity are associated with cancer. As a result, identifying active deubiquitinating enzymes that directly modulate PTEN protein stability and activity for therapeutic purposes has become a high priority for cancer researchers. Indeed, new discoveries of DUBs that interact with PTEN have changed our understanding of PTEN function and regulation. HAUSP/USP7, USP10, USP11, USP13, OTUD3, and Ataxin-3 have all been recently identified as PTEN DUBs that control PTEN activity in different cancer-specific contexts. However, these DUBs play context-dependent tumor suppressor or oncogenic roles in cancer progression, and in different contexts, both their up- and downregulation can be hallmarks of tumor cells leading to malignancies; therefore, a complete understanding of how each individual DUB functionally influences tumorigenesis or tumor suppression remains unclear, and further in vivo investigation is required. Although researchers have extensively described the specificity of PTEN DUBs, as discussed herein, their ubiquitin linkage specificity (i.e., K6, K11, K27, K29, K33, K48 or K63-linked mono- and polyubiquitin chains) with respect to PTEN is still being elucidated. In addition, whether and how the complex relationship between PTEN DUBs (e.g., HAUSP−USP10^113^, HAUSP−USP11^114^ and USP10−USP13^70^) strengthens their activity toward PTEN requires further study. It will be interesting to evaluate the possible crosstalk between deubiquitination and other posttranslational modifications, such as acetylation^115^, methylation^116^, and SUMO ylation^11^, during the control of PTEN stability and activity. Given recent discoveries revealing that distinct PTEN isoforms and active PTEN dimers are related to specific PTEN functions^3^, it will be interesting to determine whether and how the aforementioned and several other PTEN DUBs^117–119^ impact the stability, localization and biological activity of dimeric PTEN and various PTEN isoforms. PTEN is a major tumor suppressor protein whose expression and activity often serve as the bases of diagnostic and prognostic assessment; however, no available therapy that directly targets PTEN itself is currently available. With the link of DUBs to reactivated PTEN established, the pharmacological manipulation of DUBs holds great clinical promise and suggests innovative and effective therapeutic approaches.
